# *Shigella* virulence protein VirG is a broadly protective antigen and vaccine candidate

**DOI:** 10.1038/s41541-023-00797-6

**Published:** 2024-01-02

**Authors:** Girmay Desalegn, Chitradevi S. Tamilselvi, Jose M. Lemme-Dumit, Shannon J. Heine, Dylan Dunn, Esther Ndungo, Neeraj Kapoor, Edwin V. Oaks, Jeff Fairman, Marcela F. Pasetti

**Affiliations:** 1 Center for Vaccine Development and Global Health, University of Maryland School of Medicine, 685W. Baltimore Street, Baltimore, MD 21201 USA; 2 Vaxcyte, Inc., 825 Industrial Road, San Carlos, CA 94070 USA; 3 Patuxent Research and Consulting Group, 3106 Arrowhead Farm Rd, Gambrills, MD 21054 USA

**Keywords:** Protein vaccines, Preclinical research

## Abstract

Diarrhea caused by *Shigella* has been associated with high morbidity and mortality in young children worldwide. There are no licensed vaccines, and those clinically advanced have restricted coverage as they elicit serotype-specific immunity while disease is caused by multiple circulating serotypes. Our group had previously reported a close association between serum antibodies to the *Shigella* virulence factor VirG (or IcsA) and clinical protection in infected individuals. VirG is highly conserved among *Shigella* strains and appealing as a broad-spectrum vaccine candidate. In this study, we investigated the immunogenicity and protective capacity of VirG as a subunit vaccine in mice. The surface-exposed alpha (α) domain of VirG (VirGα) was produced as a recombinant protein. This region has almost identical immune reactivity to full-length VirG. Administered intramuscularly with alum, VirGα elicited robust immune responses and high protective efficacy against *S. flexneri* 2a and *S. sonnei*. Almost complete protection was afforded by VirGα given intranasally with the *E. coli* double mutant heat-labile toxin (dmLT). VirGα-specific antibodies recognized VirG expressed on live *Shigella*, and blocked *Shigella* adhesion and invasion to human colonic cells. These results show for the first time that VirGα is a promising cross-protective vaccine candidate to prevent *Shigella* infection.

## Introduction

Diarrheal diseases caused by *Shigella* species are associated with mortality and life-long disability globally, and disproportionally affect children living in poor areas and those lacking clean water and proper sanitation. The annual global death toll is ~200,000, one-third being children younger than 5 years of age^1^. Repeated infections lead to growth faltering, impaired cognitive development, and reduced life expectancy^2^. Although treatable by antibiotics and oral rehydration therapy, the rapid and widespread rise of antibiotic-resistant strains and the highly contagious nature of the disease surmise shigellosis as a major global public health concern^3,4^ for which there is no approved vaccine available.

Various *Shigella* vaccine candidates, i.e., orally delivered live attenuated or killed *Shigella* vaccines^5–11^ and parenterally administered subunit vaccines^12–18^ have been evaluated for safety and immunogenicity in humans. *Shigella* O-polysaccharide (OPS)-based conjugates, the most advanced, are being evaluated in controlled human infection models (CHIM) (NCT04078022) and field efficacy trials (NCT04602975). An early *Shigella* OPS-recombinant *Pseudomonas aeruginosa* exotoxin A (rEPA) conjugate, although effective in adults^12^ and older children^13^, failed to prevent shigellosis in children younger than 3 years of age—the most affected group^13^. A newer version of *Shigella flexneri* (*S. flexneri*) 2a OPS-rEPA was moderately protective against only severe disease in a CHIM study^14^. These vaccine candidates rely mainly on immunity that is specific to the bacterial lipopolysaccharide (LPS) and like most others, their coverage is restricted to the immunizing serotype, while there are more than 50 disease-causing *Shigella* serotypes. OPS-based multivalent formulations that would cover the four most prevalent serotypes are being studied (NCT05156528 and NCT04056117). A multivalent outer membrane vesicle-based vaccine is also being evaluated in adults, 2–5-year-old children, and infants 9 months of age in Kenya (NCT05073003), although its monovalent predecessor failed to elicit meaningful protection in a recent adult CHIM study^17^. Still, important obstacles of the multivalent vaccine approach include difficult clinical evaluation and potential interaction/interference among vaccine components that may reduce vaccine effectiveness and increase manufacturing cost.

In addition to OPS-based vaccines, highly conserved protein components of the *Shigella* type III secretion system (T3SS) have been proposed as vaccine candidates. Our group and others reported robust preclinical immunogenicity and protective efficacy of the invasion plasmid antigens (Ipa) B, alone or combined with other antigens, against infection with multiple *Shigella* serotypes^19–27^. Invaplex, a macromolecular *Shigella* subunit vaccine containing Ipa proteins and LPS, elicited high levels of protection against *S. flexneri* 2a and *S. sonnei* in preclinical studies^28,29^, and was well-tolerated and immunogenic in humans^30,31^. However, the native Invaplex vaccine failed to prevent disease in an adult CHIM^32^.

Seeking to identify a protein-based vaccine candidate that would be safe, robustly immunogenic, and broadly protective, we focused our efforts on the *Shigella* virulence antigen VirG, also known as IcsA (Intra-cellular spread gene A). VirG is integrally involved in bacterial pathogenesis; it is essential for *Shigella* adherence^33,34^ and actin-based motility within the colonic epithelium^35–37^. Disruption of *virG* largely attenuated *Shigella* virulence in humans and in animal models^10,38–40^. The protein is composed of two main domains: a functionally active surface-exposed α-domain and a transporter transmembrane β-domain^41^. Importantly, VirG is highly conserved among *Shigella* species and independent of the T3SS. High levels of VirG-specific IgG were detected in adults living in *Shigella* endemic areas^42^ who become refractory to infection. Our group has found, in several independent analyses, a strong association between antibodies to VirG and clinical protection against *S. flexneri* 2a infection in humans^43,44^. This evidence prompted us to investigate VirG as a *Shigella* vaccine candidate.

In this study, we describe the production of the N-terminal α-domain of VirG (VirGα) and its immunogenicity and protective capacity against *Shigella* infection in mice immunized via parenteral or mucosal route. VirGα elicited robust immune responses, including antibodies capable of blocking bacterial adhesion and invasion, and afforded high levels of protection against *S. flexneri* 2a or *S. sonnei*.

## Results

### Production of recombinant VirGα

**Fig. 1:**
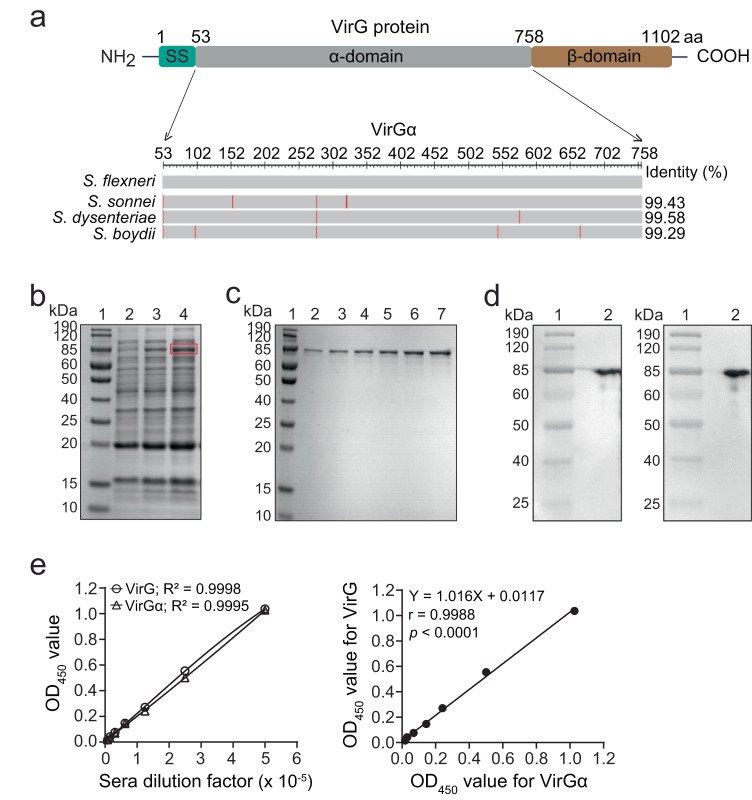
Cloning, expression, and purification of *Shigella* VirGα. **a** Schematic representation of full-length VirG and the α-domain region. SS: Signal Sequence. The VirGα aa sequences across the four *Shigella* species were determined using NCBI multiple sequence alignment viewer. Identity represents the percent homology of VirGα sequences and non-identical aa residues (red bars) in *S. sonnei*, *S. dysenteriae*, and *S. boydii* compared to *S. flexneri*. **b**–**d** SDS-PAGE gels and western blot analyses of purified VirGα. **b** Protein expression by positively transformed *E. coli* BL21 (DE3) pLysS competent cells induced with 1 mM IPTG for 2 h (lane 3) or 3 h (lane 4), the latter achieving higher expression of the 76 kDa VirGα (lane 4, red box). Protein molecular weight marker (MWM; lane 1) and uninduced cells (lane 2) were included as controls. **c** Purified VirGα after concentration; protein MWM (lane 1), eluted VirGα protein (lanes 2–5) and concentrated VirGα (lanes 6–7). **d** Confirmation of 76 kDa VirGα protein (lane 2) by western blot with anti-His tag antibody (left panel) and anti-VirGα immune sera (right panel). MWM (lane 1). **e** Immune reactivity of VirGα compared to full-length VirG determined by ELISA using pooled sera from mice immunized with full-length VirG. Serially diluted sera were tested against each antigen in identical conditions. Data represent dose–response reactivity as mean OD_450_ values from replicate wells (left panel) and correlation of immune reactivity against full-length VirG and VirGα (right panel) across serum dilutions. *R*^2^, Pearson’s *r* and *P* values are indicated.

A schematic representation of VirG regions is shown in Fig. 1a. Full-length VirG expressed poorly as a recombinant protein, likely due to its large size (120 kDa). Therefore, the surface-exposed region, VirGα (aa 53–758), was cloned, expressed, and produced as a recombinant protein in an *E. coli-*based system. The homology of the VirGα amino acid sequences among the most prevalent *Shigella* serotypes is >99% (Fig. 1a). The VirGα gene (2.1 kb) was amplified and inserted into a pRSETA plasmid and transformed into *E. coli* DH5α competent cells (Supplementary Fig. 1a, b). Restriction enzyme digestion with BamHI and EcoRI was performed to confirm successful cloning (Supplementary Fig. 1c). VirGα-bearing plasmids were transformed into *E. coli* BL21 (DE3) pLysS competent cells; bacteria were grown, and protein expression was induced. High levels of recombinant VirGα (VirGα, 76 kDa) were produced after 3 h of induction (Fig. 1b). VirGα was purified using a chelating nickel column and refolded within the column. The protein was eluted, dialyzed, and concentrated, resulting in a 76 kDa band in the sodium dodecyl sulfate-polyacrylamide gel electrophoresis (SDS-PAGE) gel (Fig. 1c). Identity was confirmed by western blot analysis using anti-His tag antibody (Fig. 1d, left panel) and VirGα-reactive mouse antisera (Fig. 1d, right panel). *E. coli*-based production of VirGα was efficient, with a recovery yield of 6.2 mg/L culture, >95% purity, <1% residual host-cell proteins, and <20 endotoxin units/mg (detection limit); these results were reproduced in subsequent batches.

The immune reactivity of VirGα was compared with that of full-length VirG using pooled sera from mice immunized with full-length VirG in an indirect ELISA. OD_450_ values produced by serially diluted sera in plates coated with each antigen (and run in identical conditions) showed linear dose–response curves (Fig. 1e, left panel) and were positively and significantly correlated with a Hill slope of 1 (*r* = 0.9988, *P* < 0.0001) (Fig. 1e, right panel). These results indicate that the immune reactive epitopes present on the full-length VirG are vastly contained in protein’s α-domain region.

### Parenteral immunization with VirGα elicits robust and dose-dependent antibody responses

**Fig. 2:**
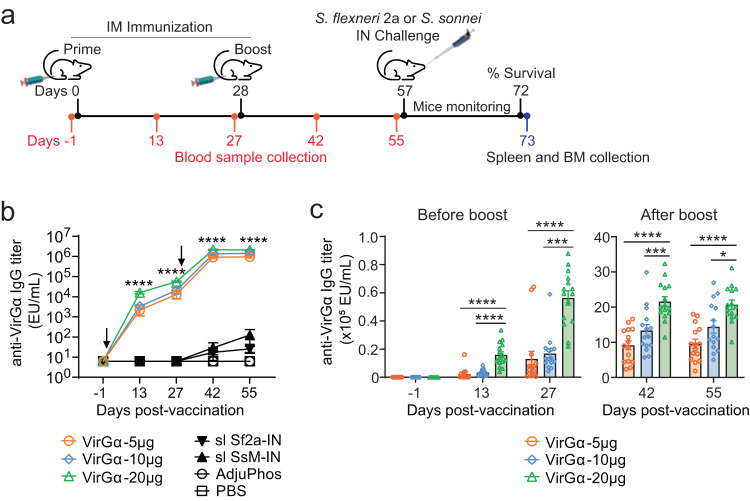
Immunogenicity of *Shigella* VirGα. **a** Schematic illustration of immunization and challenge. Adult mice (15–20/group for each challenge strain) were immunized intramuscularly (IM) on days 0 and 28 with 5, 10, or 20 µg of VirGα adsorbed to AdjuPhos®. Blood samples were collected prior- and post vaccination every two weeks thereafter (indicated in red). Negative control groups received PBS or AdjuPhos®. Mice immunized intranasally (IN) at the same timepoints with a sublethal dose (1.0×10^5^ CFU/dose) of *S. flexneri* 2a (sl Sf2a-IN) or *S. sonnei* Moseley (sl SsM-IN) were included as positive controls for *S. flexneri* 2a 2457T or *S. sonnei* Moseley challenge, respectively. BM, bone marrow. **b** Kinetics of VirGα-specific serum IgG titers measured by ELISA (*n* = 15/group); arrows indicate immunization. Data represent mean of individual titers ± SEM; ****P < 0.0001 vs. PBS or AdjuPhos® by *t* test. **c** VirGα-specific IgG titers achieved before and after boost with escalating doses of VirGα (*n* = 15/group). Data represent mean of individual titers ± SEM; *P < 0.05, ***P < 0.001, and ****P < 0.0001 by one-way ANOVA with Tukey’s multiple comparisons test.

To investigate the immunogenicity of VirGα, adult mice were immunized intramuscularly (IM) with escalating doses (5, 10, or 20 µg) of VirGα admixed with AdjuPhos® on two occasions (day 0 and 28) as illustrated in Fig. 2a. Negative control groups received AdjuPhos® or phosphate-buffered saline (PBS). Mice immunized intranasally (IN) with sublethal doses (1 × 10^5^ CFU/dose) of *S. flexneri* 2a or *S. sonnei* were included as positive controls of serotype-specific protection. Parenteral immunization with VirGα elicited high levels of VirGα-specific serum IgG in all vaccinated groups compared to PBS or AdjuPhos® control (Fig. 2b). Antibody responses increased after the second immunization, attaining peak levels 2 weeks after the boost in all groups (Fig. 2b). VirGα-specific IgG responses exhibited a dose–response pattern, i.e., the magnitude of serum IgG was higher in groups that received larger amounts of VirGα at all timepoints post vaccination (Fig. 2c). The best responders were mice that received the 20 µg dose; mean serum IgG titers at day 27 (before boost) were 1.3 × 10^4^, 2.1 × 10^4^, and 5.6 × 10^4^ ELISA Units (EU)/mL in groups that received 5, 10, and 20 µg, respectively, and 9.6 × 10^5^, 1.4 × 10^6^, and 2.1 × 10^6^ EU/mL at day 55 (after boost and around the time of challenge) (Fig. 2b, c). Mean fold increases in serum IgG titers over baseline at day 55 amounted to 153,600, 224,000, and 336,000 EU/ml for the 5, 10, and 20 µg dosage levels, respectively (Fig. 2b, c). An unexpected finding was the low (negligible) VirGα-specific serum IgG responses in mice immunized IN with sublethal doses of *S. flexneri* 2a and *S. sonnei* (Fig. 2b), while these controls developed robust serum IgG responses to the OPS of the respective immunizing strain^24^. VirGα-specific IgA responses were detected neither in serum or feces of mice immunized with VirGα via IM, nor in those that received sublethal IN doses of *S. flexneri* 2a and *S. sonnei* (data not shown).

### VirGα protects mice from *S. flexneri* 2a and *S. sonnei* lethal infection

**Fig. 3:**
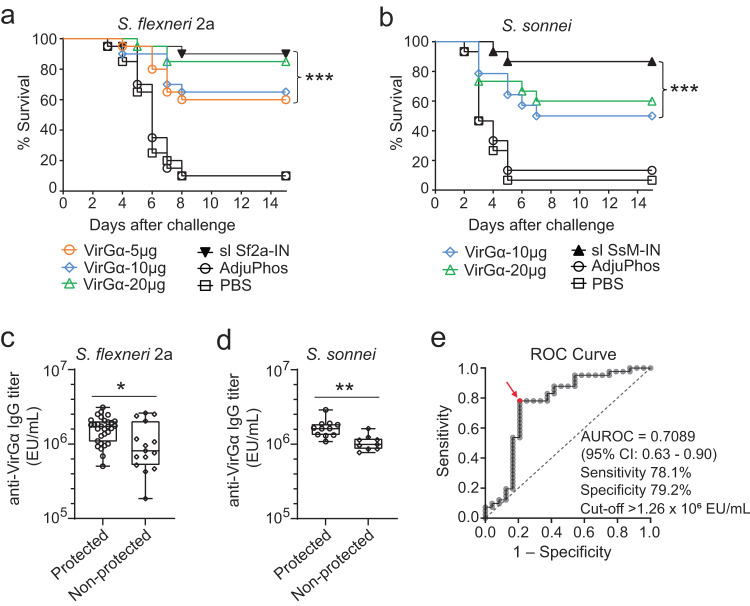
Protective efficacy of *Shigella* VirGα. **a**, **b** Mice were immunized as described in Fig. 2 and challenged IN with a lethal dose of *S. flexneri* 2a 2457T (5.0 × 10^6^ CFU/dose) or *S. sonnei* Moseley (5.2 × 10^6^ CFU/dose) on day 57 post vaccination. Survival curves from 15 to 20 mice/group are shown. *P* values compared to PBS control were determined by log-rank (Mantel–Cox) test. **c**, **d** VirGα-specific serum IgG titers at the time of challenge (day 55) in VirGα vaccinated mice that were protected or succumbed to *S. flexneri* 2a (*n* = 45) or *S. sonnei* (*n* = 20) challenges. Individual data points and box plots indicating median, quartiles, minimum and maximum values are shown, *P < 0.05 and **P < 0.01 by *t* test. **e** ROC analysis of VirGα-specific serum IgG levels in VirGα vaccinated survivors vs. those that succumbed to *Shigella* infection (*n* = 65 combining both challenges) and identification of a protective threshold. Red arrow denotes the antibody level predictor of survival with ~80% sensitivity and specificity. AUROC = 0.7089 (95% CI: 0.63–0.90), *P* = 0.0005.

Mice immunized IM with increasing doses of VirGα (as described above) were subjected to lethal IN challenge with virulent *S. flexneri* 2a 2457T (5.0 × 10^6^ CFU/dose) or *S. sonnei* Moseley (5.2 × 10^6^ CFU/dose) on day 57 post vaccination (Fig. 2a). Parenteral immunization with VirGα adjuvanted with AdjuPhos® afforded high levels of protection against *S. flexneri* 2a; 83% vaccine efficacy (VE) was achieved with the 20 µg dose, followed by 61% and 56% VE with 10 and 5 µg, respectively (Fig. 3a and Table 1). The VE was significantly higher in all vaccinated groups as compared to the negative control (*P* < 0.001) but did not reach statistical significance when compared among VirGα vaccinated groups. Importantly, VirGα conferred statistically significant cross-protection against *S. sonnei* lethal infection; the 20 µg and 10 µg doses achieved 57% and 46% VE, respectively (Fig. 3b and Table 1). The lower VE of the 20 µg and 10 µg dose of VirGα against *S. sonnei* is attributed to a more severe infection produced by *S. sonnei* Moseley in this model (mice succumbed sooner). Mice immunized IN with sublethal doses of virulent *S. flexneri* 2a or *S. sonnei* afforded the highest homologous protection, although not significantly different compared to that afforded by 20 µg of VirGα. Virtually all unvaccinated control mice succumbed to lethal pulmonary *Shigella* challenges (Fig. 3a, b).

**Table 1:** Efficacy of VirGα-AdjuPhos® administered IM against *Shigella* infection.

*S. flexneri* 2a			*S. sonnei*		
Vaccine^c^	% VE^a^	*P* value^b^	Vaccine^c^	% VE^a^	*P* value^b^
VirGα—5 µg	55.6	0.0002	VirGα-10µg	46.4	0.0018
VirGα—10 µg	61.1	<0.0001	VirGα-20µg	57.1	0.0009
VirGα—20 µg	83.3	<0.0001	sl SsM-IN	85.7	<0.0001
sl Sf2a-IN	88.9	<0.0001	AdjuPhos®	7.1	0.6656
AdjuPhos®	0.0	0.7596	PBS		
PBS					

^a^% Vaccine efficacy (VE) = [(% death in PBS control mice − % death in vaccinated mice)/% death in PBS control mice] × 100.

^b^Survival curves of vaccinated groups were compared to PBS using the log-rank (Mantel–Cox) test.

^c^sl Sf2a-IN, sublethal dose of *S. flexneri* 2a-IN; sl SsM-IN, sublethal dose of *S. sonnei* Moseley-IN.

Based on the associations between antibody levels and reduced infection we had observed in humans, we investigated whether the level of serum IgG elicited by vaccination with VirGα was associated with mouse survival post infection. VirGα immunized mice that were protected from *S. flexneri* 2a or *S. sonnei* challenge had significantly higher levels of VirGα-specific serum IgG at the time of challenge (at day 55) as compared to those that did not survive (non-protected) (Fig. 3c, d). By applying receiver operating characteristics (ROC) curve and area under curve (AUC) analyses, we identified a threshold level (1.26 × 10^6^ EU/mL) that would allow the prediction of protective outcome with ~80% sensitivity and specificity (Fig. 3e).

**Fig. 4:**
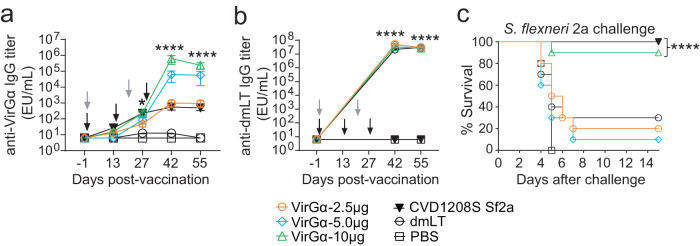
Immunogenicity and protective efficacy of VirGα administered mucosally. Adult mice (10 per group) were immunized IN with 2.5, 5, or 10 μg of VirGα admixed to dmLT (2.5 μg) on days 0, 14, and 28 (black arrows). Negative control mice received PBS or dmLT. Mice immunized IN with CVD 1208S on days 0 and 21 (gray arrows) were included as positive controls for *S. flexneri* 2a challenge. **a** Kinetics of VirGα-specific serum IgG titers measured by ELISA. Mean of individual titers ± SEM; *P < 0.05, ****P < 0.0001 vs. PBS by Mann–Whitney test. **b** Kinetics of dmLT-specific serum IgG measured by ELISA. Mean of individual titers ± SEM; ****P < 0.0001 compared to PBS control by *t* test. **c** Immunized mice were challenged IN with 1 × 10^7^ CFU/dose of *S. flexneri* 2a on day 56 post vaccination. Data represent survival curves from ten mice per group. ****P < 0.0001 compared to PBS control as determined by log-rank (Mantel–Cox) test.

The immunogenicity and protective efficacy of VirGα were also investigated using a mucosal route and a clinically advanced mucosal adjuvant, the *E. coli* double mutant heat-labile toxin (dmLT). Mice were immunized IN with 2.5, 5, or 10 µg of VirGα admixed with dmLT on days 0, 14, and 28; this schedule was selected based on results from previous studies^19,45^. VirGα-specific serum IgG responses after the 1st and 2nd vaccinations were modest but increased dramatically after the 3rd immunization. The 10-µg dosage achieved the highest titers (Fig. 4a) and afforded a VE of 90% against *S. flexneri* 2a; the VE values for the 5 or 2.5 µg immunizing doses were negligible (Fig. 4c and Table 2). A strict minimum amount of VirGα + dmLT (10 µg) was required to achieve protection via IN immunization. High levels of dmLT-specific IgG titers were observed that were almost identical among dmLT recipient groups, indicating proper vaccine preparation and immunization (Fig. 4b).

**Table 2:** Efficacy of VirGα-dmLT administered IN against *Shigella flexneri 2a* infection.

*S. flexneri* 2a		
Vaccine^c^	% VE^a^	*P* value^b^
VirGα—2.5 µg	20.0	0.0594
VirGα—5 µg	10.0	0.5868
VirGα—10 µg	90.0	<0.0001
CVD 1208S Sf2a	100.0	<0.0001
dmLT	30.0	0.2194
PBS		

^a^% Vaccine efficacy (VE) = [(% death in PBS control mice − % death in vaccinated mice)/% death in PBS control mice] × 100.

^b^Survival curves of vaccinated groups were compared to PBS control using the log-rank (Mantel–Cox) test.

^c^Sf2a, S. flexneri 2a.

### VirGα-specific antibody-secreting cells (ASC) in systemic tissues

The frequencies of IgG and IgA ASC were measured in spleen and bone marrow from mice immunized IM with 20 µg of VirGα (the most efficacious dose) and from mice inoculated IN with sublethal doses of *S. flexneri* 2a (Sf2a-IN). This analysis was conducted in animals that survived the *S. flexneri* 2a lethal infection to discern vaccine-elicited immunity evoked by infection. VirGα-alum IM immunization was chosen for this and subsequent studies as this mode of vaccination is the most likely to be used in humans.

**Fig. 5:**
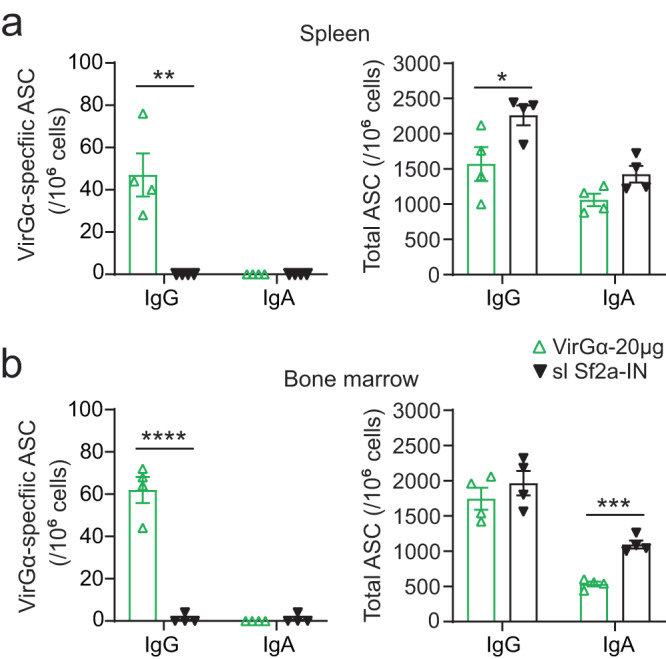
VirGα-specific and total ASC. **a**, **b** Spleen and bone marrow were obtained from mice immunized IM with 20 µg of VirGα (IM) or IN with *S. flexneri* 2a (sl Sf2a-IN) that survived *S. flexneri* 2a lethal challenge. Single-cell suspensions from individual mice in each group were pooled, and VirGα-specific and total IgG and IgA ASC were measured by ELISpot in quadruplicates. Bars represent mean values per 10^6^ cells ± SEM; *P < 0.05, **P < 0.01, ***P < 0.001, and ****P < 0.0001 by *t* test.

High levels of VirGα-specific IgG ASC were detected both in the spleen and bone marrow of VirGα vaccinated mice (Fig. 5a, b). In contrast, VirGα-specific IgG ASC were not detected in mice that received Sf2a-IN. The absence of VirGα-specific IgG ASC in the Sf2a-IN mice is consistent with their negligible antibody responses to VirGα (Fig. 2b). All groups exhibited high frequencies of total (vaccine-agnostic) IgG in the spleen and bone marrow. In fact, mice that received Sf2a-IN had total IgG ASC levels even higher than the VirGα-immunized mice ruling out a technical error (Fig. 5a, b). Neither mice that received VirGα nor the positive control immunized with sublethal Sf2a-IN developed VirGα-specific IgA ASC (Fig. 5a, b); meanwhile, total IgA ASC were detected in spleen and bone marrow from both groups. The absence of VirGα IgA ASC in these groups is consistent with the lack of serum and fecal IgA responses described above.

### Antimicrobial function of VirGα-specific antibodies

**Fig. 6:**
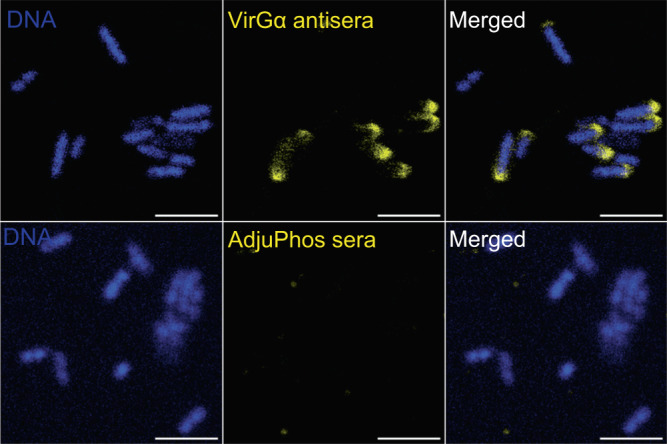
*Shigella* VirG recognition by VirGα-induced antibodies. *S. flexneri* 2a in suspension were incubated with pooled sera from VirGα-vaccinated mice (top panel) or mice that received AdjuPhos® (bottom panel). Bound antibodies were detected using AF555-labeled anti-mouse antibodies. Bacteria were mounted in antifade reagent with DAPI for DNA staining. Confocal images were captured with a 60× water immersion objective. Scale bar = 5 µm.

The association between serum antibody levels and protection prompted us to examine the antimicrobial mechanism of VirGα antibodies. To this end, we first investigated the capacity of VirGα mouse immune sera to recognize VirG expressed on live *S. flexneri* 2a by confocal immunofluorescence microscopy. Vaccine-induced VirGα-specific antibodies, but not AdjuPhos® antisera, recognized VirG expressed by *S. flexneri* 2a in its typical unipolar pattern, as shown by confocal microscopy images (Fig. 6).

**Fig. 7:**
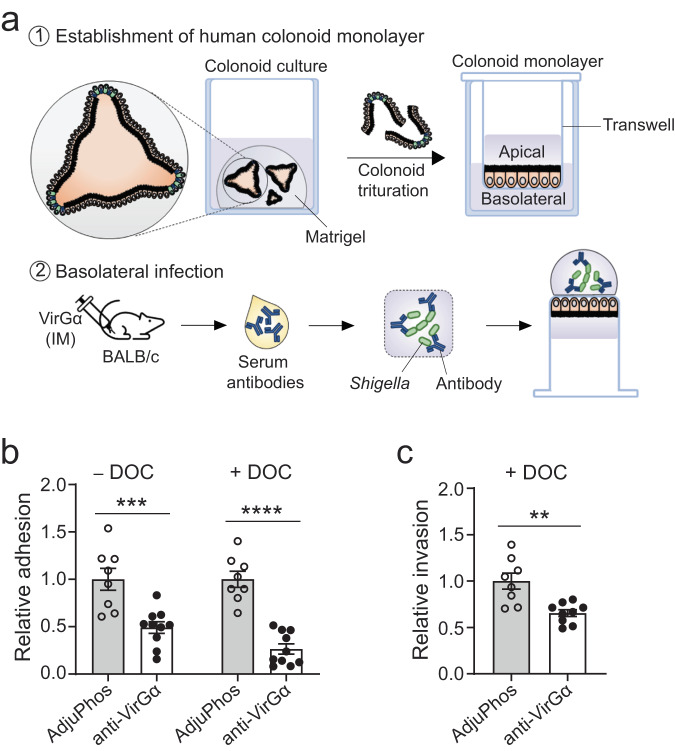
VirGα antibodies reduced *Shigella* adherence and invasion to human colonoid monolayers. **a** Illustration of the human colonoid infection model. *S. flexneri* 2a grown in the absence or presence of DOC and pre-incubated with VirGα mouse antisera were added to human colonoid monolayers from the basolateral side, as described in “Methods”. Pooled sera from mice that received AdjuPhos® was used as a negative control. **b** Adherence inhibition. **c** Invasion inhibition. Relative adhesion or invasion of *S. flexneri* 2a in the presence of anti-VirGα sera was determined in comparison to that of the negative control (normalized to 1). Assays were performed in at least quadruplicate wells, and two independent experiments were conducted. Data represent mean from replicate wells ± SEM; **P < 0.01, ***P < 0.001, ****P < 0.0001 by *t* test.

We next interrogated the capacity of immune sera from VirGα vaccinated mice to block *Shigella* adherence to intestinal epithelial cells using primary human colonoid monolayers^46–48^. *S. flexneri* 2a admixed with VirGα-specific murine antisera (or sera from mice that received AdjuPhos® as negative control) were added to the basolateral side of human colonoid monolayers (Fig. 7a). VirGα-specific immune sera inhibited *Shigella* adherence to human colonoids; a statistically significant ~50% reduction in bacterial adhesion was observed (Fig. 7b). The inhibition of adherence by VirGα-specific antibodies was more pronounced (only 26% of organisms remained attached) when the bacteria were grown in the presence of deoxycholate (DOC) (Fig. 7b), a bile salt that enhances the ability of *Shigella* to attach to and invade epithelial cells^34,49^ and activates VirG-dependent *S. flexneri* adhesion^33^. Likewise, VirGα-specific antibodies reduced adherence of *S. sonnei* to human colonoids (Supplementary Fig. 2). Using the same approach, we also demonstrated the capacity of VirGα-specific mouse antisera to reduce colonocyte invasion by DOC-stimulated *S. flexneri* 2a; only 65% of the original bacterial load was recovered intracellularly (Fig. 7c). These results demonstrate the capacity of vaccine-induced VirGα-specific IgG to recognize the native antigen in living organisms and to deploy antimicrobial functions impairing bacterial adhesion and invasion of human colonocytes.

## Discussion

*Shigella* dysentery is the main cause of bacterial diarrheal death globally and a safe, effective, and affordable vaccine remains a critical need and a public health priority^50^. A major disadvantage of the vaccine candidates most advanced in clinical programs (i.e., *Shigella* OPS glycoconjugates) is their restricted coverage—only against the immunizing serotype, despite the many disease-causing serotypes. Multivalent glycoconjugate formulations have been proposed to extend coverage to the most prevalent serotypes. However, this approach has another set of challenges: potential interference among vaccine antigens, hyperimmunization with carrier proteins (some of which are already used in vaccines given routinely to children of target age), challenging clinical development and regulatory pathways, increased complexity in manufacturing, and higher costs.

In this study, we report *Shigella* VirGα as a new subunit-based vaccine candidate; VirGα exhibited robust immunogenicity and broad protective capacity. Parenteral immunization of mice with VirGα adjuvanted with alum, chosen for its safety record in routine pediatric immunization^51^, elicited vigorous serum antibody responses and afforded > 80% protection against *S. flexneri* 2a and 57% against *S. sonnei* lethal infection. Similarly, mucosal (IN) immunization with VirGα admixed with *E. coli* dmLT, a clinically advanced mucosal adjuvant^52^, conferred 90% protection against *S. flexneri* 2a infection. VirGα serum IgG produced by parenteral immunization was associated with vaccine effectiveness. A threshold level was identified as a predictor of protective efficacy in our experimental setting; a similar value was reported for IpaB^22^. The concordance between VirG-specific immunity and protection in mice is consistent with the association we had observed between the magnitude of serum IgG (IgG1) against VirG and reduced disease severity in volunteers experimentally challenged and re-challenged with *S. flexneri* 2a^43,44^. It is also in agreement with the high levels of VirG-specific antibodies we found in adults living in *Shigella* endemic areas^42^, who are less susceptible to infection than children, due to natural immunity acquired through repeated exposure. Similarly, we found VirG-specific IgG and IgA in stool from 0- to 5-year-old children infected with *Shigella*, and these antibodies were more abundant in children who did not develop dysentery as compared with those who had dysentery or diarrhea from other causes^53^. Taken together, these observations point to VirG as an important target of protective immunity, and VirG-specific antibodies as putative antimicrobial effectors.

We confirmed that VirGα antibodies from vaccinated mice recognized VirG expressed on the bacterial outer surface and demonstrated their capacity to prevent *Shigella* infection by blocking bacterial adhesion and invasion in human colonoid monolayers. The functional recognition of *Shigella* VirG by VirGα-specific antibodies confirms VirGα’s proper conformation and preservation of immunogenic epitopes. These results indicate that VirG antibodies display critical antimicrobial functions that can prevent *Shigella* infection in the mucosal interface, averting tissue damage and inflammation. In the context of interrogating bacterial pathogenesis, purified VirG-specific antibodies were shown to block the attachment of a hyperadhesive ∆IpaD *Shigella* mutant to mammalian cells^34^, which is consistent with our findings. In a recent system serology study encompassing a broad analysis of antibody binding and functional features, we probed the immunoglobulin class and subclass distribution and Fc receptor binding profile of VirGα-specific antibodies, as well as their capacity to engage innate immune cells using sera obtained from individuals who participated in a CHIM study^44^. VirGα-specific IgG and IgA, Fc receptor binding, as well as VirG-specific antibody-dependent complement deposition (ADCD) and antibody-dependent cellular monocyte phagocytosis (ADCP) were identified in individuals who experienced minimal disease^44^.

In addition to systemic antibodies, IM delivered VirGα elicited antigen-specific IgG ASC that were detected in the spleen and bone marrow. Vaccine-induced ASC, along with memory B cells, provide long-term humoral immunity to infection. Spleen ASC derived from germinal center B cells egress to blood and replenish circulating antibody levels. They can also migrate to bone marrow. The ASC in bone marrow represent long-lived plasma cells that maintain humoral immune memory^54^. VirGα-specific humoral and ASC responses were IgG-rich, which is expected for subunit vaccines given parenterally in the presence of alum. A predominant IgG response is also elicited by subunit vaccines in routine immunization programs. The negligible ASC and antibody responses to VirGα in mice dosed IN with live *Shigella* (either a live attenuated strain or wild-type organisms) was surprising and intriguing, and we propose could be attributed to limited antigenic exposure—insufficient to adequately stimulate immune responses. Surface expression of VirG in *Shigella* is mainly confined to one pole and masked by LPS^55^. Adult individuals living in areas where *Shigella* is endemic have high prevalence of VirG-reactive antibodies but natural immunity in these individuals is acquired through life-long exposure. The poor immunogenicity of VirGα in mice infected IN as compared to humans infected orally may also reflect differences in host susceptibility and immunity between species and routes of infection. *Shigella* spp. do not infect rodents via the oral route^56^, and the pulmonary challenge is only a proxy for shigellosis in humans^57^.

We and others have investigated the suitability of conserved proteins that comprise the *Shigella* T3SS (i.e., IpaB, IpaC, and IpaD) as broad-spectrum subunit vaccine candidates^43,58^. Investigators at Walter Reed Army Institute of Research (WRAIR) sought a combination of *S. flexneri* 2a LPS and recombinant IpaB and IpaC (Invaplex_AR-DETOX_) which is in clinical evaluation^32^. A polyvalent fusion protein containing epitopes from IpaB, IpaD, VirG, GuaB and Shiga toxins has been shown to elicit broadly functional antibodies and cross-protection against *S. sonnei* and *S. flexneri* serotypes^27^. Our group developed a *Shigella* OPS-IpaB conjugate vaccine candidate capable of eliciting cross-protective immunity in mice^24^. IpaB and VirG are appealing vaccine targets because of their immunogenic properties and association with protective immunity in humans^43,44,58^.

Conceivably, VirGα could operate as a stand-alone vaccine or could be combined with IpaB to maximize efficacy. A vaccine based on broad-spectrum safe and effective proteins would be simpler and superior to existing candidates in their greater breadth of coverage, ability to prevent infection caused by eventually all serotypes, ease of manufacturing and quality control, straightforward clinical evaluation, and practical implementation. These advantages will reduce cost and make the vaccine more affordable and appealing to limited-resource countries. Based on the excellent safety profile of subunit vaccines in routine schedules, a protein-based *Shigella* vaccine is expected to be well-tolerated by infants and young children, who experience the highest burden of disease. The remarkable immunogenicity of VirGα (the same for IpaB) is propitious to generate robust and long-lasting protective immunity in those under three years of age for whom *Shigella* OPS-rEPA^13^ and orally delivered live attenuated vaccines^59^ have failed. The uncomplicated production of a subunit vaccine will increase affordability. Implementation of such a vaccine, even in poor-resource areas, is achievable by incorporation into existing schedules. The overall disease prevention strategy using broad protective proteins is simple and anticipated to be cost-effective. As such, a VirGα or VirGα + IpaB vaccine meet the WHO-preferred product features outlined for a vaccine against *Shigella*^50^.

The cross-protective advantage of conserved proteins must be emphasized as circulation of disease-causing serotypes in the environment is cyclical, and the emergence of strains not contained in O-antigen-based vaccines would require product reconfiguration. The landscape of *Shigella* vaccines will likely change in the next 5 years as refined concepts and new candidates advance to human clinical trials and CHIM^60^. Plans to evaluate the safety and efficacy of VirG and IpaB-containing vaccines are underway.

In conclusion, the results presented herein demonstrate the capacity of *Shigella* VirGα to elicit vigorous immune responses to prevent disease caused by multiple serotypes. We showed for the first time that VirGα is a promising cross-protective *Shigella* vaccine candidate. The concept of a broad protective subunit vaccine is simple and easy to implement. Vaccine features, including preclinical VE, meet the WHO-preferred product profile for a *Shigella* vaccine^50^.

## Methods

### Bacterial growth

*S. flexneri* 2a 2457T was grown in Luria-Bertani (LB) media (Athena Environmental Sciences, Baltimore, MD) at 37 °C, and the invasion plasmid was extracted using a plasmid purification kit (Qiagen, Germantown, MS). *E. coli* DH5α and BL21 (DE3) pLysS cells were cultured in LB media at 37 °C. *S. flexneri* 2a 2457T and *S. sonnei* Moseley were prepared as previously described^19,24^. Briefly, the strains were streaked on tryptic soy agar (TSA) containing 0.02% Congo red and grown overnight at 37 °C. A total of 20–25 colonies were picked and grown in 125 mL LB or tryptic soy broth (TSB) at 37 °C with agitation (180 rpm) for 2–3 h or until an OD_600_ of 0.8 to 1.3 was achieved. Bacterial cultures were centrifuged, and pellets were resuspended in PBS. CFU were determined by plating serial dilutions of bacteria suspension on TSA and/or Congo red agar plates.

### Cloning, expression, and purification of recombinant VirGα

The gene for expression of VirGα (amino acid [aa], GenBank accession number AF386526.1 from *S. flexneri* 2a) was synthesized and subcloned in pRSETA vector. VirGα gene (2.1 kb) was amplified by PCR using as a template the 220 kb *S. flexneri* 2a 2457T virulence plasmid. The amplified VirGα gene was purified using the gel extraction kit (Qiagen). pRSETA, a high-level prokaryotic expression vector was used to clone VirGα gene with a cleavable poly-histidine (6x His) tag which enables rapid purification with nickel resin and detection with an anti-His antibody. The purified VirGα gene and pRSETA expression vector was digested with restriction enzymes BamHI and EcoRI, followed by gel elution and purification. The digested VirGα and pRSETA vector were ligated and transformed into *E. coli* DH5α competent cells. After transformation, the colonies bearing VirGα-encoding plasmids were screened by PCR and ran on 1% agarose gel. The plasmids containing the VirGα gene were extracted using plasmid isolation kit (Qiagen). Restriction enzyme digestion with BamHI and EcoRI was performed to further confirm VirGα gene cloning. The plasmid-bearing strains were stored in 15% glycerol stock at −80 °C.

Plasmids bearing VirGα were transformed into *E. coli* BL21 (DE3) pLysS cells for protein expression. *E. coli* cells were grown in 8 L of Terrific broth (Invitrogen) with 30 µg/mL chloramphenicol and 50 µg/mL ampicillin at 37 °C, 200 rpm, until reaching an OD_600_ of 4.0–5.0. Protein expression was induced with 1 mM isopropyl 1-thio-β-D-galactopyranoside (IPTG) (Teknova, Hollister, CA) for 3 h. After induction, cells were centrifuged at 7800 rpm for 30 min at 4 °C. The bacterial pellet was collected and resuspended in B-PER (Thermo Fisher Scientific, Waltham, MA) extraction buffer with Protease inhibitor cocktail (Sigma-Aldrich, St. Louis, MO), Lysozyme 1 mg/mL, Benzonase 25 U/mL, and 0.5% Triton X-100 (Sigma-Aldrich) and incubated for 1 h at room temperature (RT). The suspension was then centrifuged at 10,000×*g* for 40 min, 4 °C. The pellet containing inclusion bodies was washed and solubilized in a solution containing 20 mM Tris, 0.5 M NaCl, 20 mM Imidazole, and 8 M Urea. The expressed recombinant VirGα protein was purified using a chelating nickel column (Cytiva, Marlborough, MA) by affinity chromatography. The bound protein was washed and then refolded within the column using urea gradient starting at 6 M urea in elution buffer containing 20 mM Tris, 0.5 M NaCl, 20 mM Imidazole, pH 8.0, and ending at 0 M urea in the same buffer. The refolded protein was collected in elution buffer, and urea was removed by dialysis. The purified protein was then concentrated using Amicon filters (Millipore). Protein concentration was determined using Bicinchoninic Acid (BCA) protein assay (Pierce™ Protein Assay Kit; Thermo Fisher Scientific™) with bovine serum albumin (BSA) as standard. Aliquots were stored at −80 °C. The purity of VirGα was determined based on the relative intensity and size of the bands by Image Lab^TM^ software (Bio-Rad). The endotoxin level was analyzed by limulus amoebocyte lysate (LAL) cartridges (Charles River Laboratories, Wilmington, MA) according to the manufacturer’s instruction. Residual host-cell proteins were determined based on the density of non-specific bands in SDS-PAGE with Coomassie staining. VirGα aa sequences of *S. flexneri* (NCBI reference sequence: WP_172507392.1), *S. sonnei* (WP_001071793.1), *S. dysenteriae* (WP_134801816.1) and *S. boydii* (WP_148722086.1) were used for determination of multiple sequence alignment.

### SDS-PAGE and western blot analysis

For SDS-PAGE, proteins were prepared in 2×-Laemmli sample buffer (Bio-Rad, Philadelphia, PA) added with 5% β-mercaptoethanol and denatured by heating at 95 °C for 5 min. The denatured protein samples (20 µl) were loaded onto 12% precasted polyacrylamide gels (Bio-Rad). For western blot analysis, SDS-PAGE-run samples were then transferred into the nitrocellulose membrane using Trans-Blot Turbo Transfer Packs (Bio-Rad). Then the membrane was blocked with PBS containing 0.5%Tween-20 (PBS-T) and 3% BSA, and incubated with anti-His tag antibodies (Catalogue # MA1-21315, Invitrogen; diluted 1:5,000 in PBS-T) or murine anti-VirGα immune sera (pooled from mice immunized with VirGα protein; diluted 1:1000 in PBS-T) overnight at 4 °C. The membrane was washed with PBS-T and incubated with Horseradish Peroxidase (HRP)-labeled goat anti-mouse IgG (Catalogue # 5220-0460, KPL SeraCare, Gaithersburg, MD) diluted 1:10,000 in PBS-T for 1 h at RT. The membrane was again washed with PBS-T, revealed with Immobilon Western Chemiluminescent HRP Substrate (Millipore Sigma, Burlington, MA) and analyzed using Gel Doc imaging system (Bio-Rad). Blots and gels derived from the same or side-by-side experiments were processed together. The uncropped and unprocessed images used to generate Fig. 1b–d are shown in Supplementary Fig. 3.

### Ethics statement

All animal studies and procedures were approved by the University of Maryland School of Medicine Institutional Animal Care and Use Committee (IACUC) and conducted in accordance with guidelines from the “Guide for the Care and Use of Laboratory Animals” of the National Institutes of Health (NIH). Every effort possible was made to minimize pain and distress of the animals.

### Mice, immunizations, and experimental infection with virulent organisms

Adult female BALB/c mice (6–8 weeks old) were purchased from Charles River Laboratories. For IM vaccination, mice (15–20 per group for each challenge strain) were immunized on days 0 and 28 with 5, 10, or 20 µg of VirGα adsorbed to AdjuPhos® (4.8% v/v; InvivoGen, San Diego, CA) administered in a 100 μL volume (50 μL per leg). Negative control groups received AdjuPhos® or PBS alone. Positive control groups were immunized IN on days 0 and 28 with sublethal doses (1 × 10^5^ CFU/dose) of *S. flexneri* 2a 2457 T or *S. sonnei* Moseley. For IN vaccination, mice were immunized on days 0, 14, and 28 with 2.5, 5, or 10 µg of VirGα in the presence of 2.5 µg of the *E. coli* dmLT^19^ kindly provided by PATH. Negative control groups received 2.5 µg of dmLT or PBS alone. Positive control mice were immunized IN with live attenuated strain CVD 1208S^9^ on days 0 and 21. Four weeks after the last immunization, mice were challenged IN with 0.5–1 × 10^7^ CFU/dose of *S. flexneri* 2a 2457T, corresponding to 3.5–7 50% lethal doses (LD_50_) or with 5.2 × 10^6^ CFU/dose of *S. sonnei* Moseley corresponding to 2.4 LD_50_. Blood collection and challenges were performed under Isoflurane anesthesia (with oxygen) dispensed through a precision vaporizer (VetEquip, Inc., Pleasanton, CA). Infected mice were monitored daily for 15 days as previously described^19,24^. Animals overtly sick with >20% weight loss were promptly euthanized. Mice that exhibited >20% weight loss but looked otherwise healthy were observed for 72 h and euthanized if they failed to regain weight. Mice humanely euthanized were considered non-survivors. Euthanasia was performed by CO_2_ asphyxiation followed by cervical dislocation. Blood was collected at day -1 (pre-vaccination) and days 13, 27, 42, and 55 (post vaccination) from retro-orbital or submandibular veins. Spleen and bone marrow cells were obtained from survivors (postmortem) on day 73 (day 16 post-challenge).

### Antibodies and ASC

Serum VirGα-specific IgG were measured by ELISA as previously described^19,43^. Immulon 2HB plates (Thermo Fisher Scientific) were coated with VirGα (2 µg/mL) or full-length VirG^43^ (2 µg/mL), and serum samples were tested in two-fold serial dilutions, in duplicate. Horseradish peroxidase (HRP)-labeled goat anti-mouse IgG (Catalogue # 5220-0460, KPL SeraCare, Gaithersburg, MD) diluted 1:1000 in PBS-T containing 10% nonfat dry milk was used as detection antibody. Endpoint titers were calculated by interpolation of absorbance values of samples in the linear regression curve of a calibrated in-house standard and reported as EU/mL. An EU corresponds to the reciprocal serum dilution resulting in an absorbance value of 0.2 at 450 nm above the background.

For ASC measurements, single-cell suspensions were prepared from the spleen and bone marrow of individual mice (8 per group). The spleen was disrupted with a sterile syringe plunger, and femurs and tibias were flushed by centrifugation using adaptor tubes. Red blood cells were removed with lysis buffer (BioLegend, San Diego, CA), and cell suspensions were filtered through a 70-µm cell strainer. The frequencies of total and VirGα-specific IgG- and IgA-secreting cells were determined using a mouse IgA/IgG double-color ELISpot assay kit [Catalogue # mIgGIgA-DCE-1M/2, ImmunoSpot® ELISpot Kit, Cellular Technology Limited (CTL), Cleveland, OH] according to the manufacturer’s instructions. Polyvinylidene Fluoride (PVDF) membrane ELISpot plates were pre-wet with 70% ethanol, coated with either anti-mouse Igκ/λ capture antibodies (diluted 1:100 in kit diluent A) or VirGα (2 µg/mL) overnight at 4 °C and blocked with complete RPMI1640 medium containing 10% fetal bovine serum (FBS), 2 mM l-glutamine, 100 U/mL penicillin, 100 µg/mL streptomycin, 8 mM HEPES, and 50 µM 2-mercaptoethanol for 1 h at RT. PBS-coated wells served as negative controls. Spleen and bone marrow cell suspensions from individual mice within each group were pooled, added to plates, and incubated for 6 h at 37 °C. After washing, anti-mouse IgA/IgG detection antibody (diluted 1:1000 in kit diluent B) was added, and plates were incubated in the dark at RT for 2 h. After washing, plates were again incubated for 1 h with Tertiary solution. CTL-TrueBlue and -TrueRed substrate solutions were added sequentially for color development. Spots were counted using a CTL ImmunoSpot® Analyzer along with ImmunoSpot® Software, and results were expressed as the number of spot-forming cells (SFC) per 10^6^ cells from quadruplicate wells.

### Confocal analysis of *Shigella* VirG antibody recognition

*S. flexneri* 2a were grown as described above, and a 100 µL suspension (10^7^ CFU/mL) was added to microscope chamber slides. Bacteria were fixed in aqueous 4% paraformaldehyde for 45 min at RT and then washed with PBS. After washing, fixed bacteria were permeabilized and blocked for 1 h at with PBS containing 15% FBS, 2% BSA, and 0.1% Saponin (all from Sigma-Aldrich), then rinsed with PBS and incubated overnight at 4 °C with pooled sera from mice immunized IM with VirGα or AdjuPhos® diluted 1:100 in PBS containing 15% FBS and 0.2% BSA. Stained bacteria were washed twice with PBS and incubated with AF555-labeled goat anti-mouse IgG (Catalogue # A32727, Thermo Fisher Scientific) diluted 1:100 in PBS for 1 h at RT in the dark. After washing, bacteria were mounted in ProLong Gold antifade reagent with 4,6-diamidino-2-phenylindole (DAPI) (Cell Signaling Technology, Danvers, MA) for DNA staining at 4 °C for at least 24 h. Confocal images were obtained using a MICA microscope (Leica, Wetzlar Germany). Images were captured with a 60× water immersion objective, and settings were adjusted to optimize the signal. Images were collated using FIJI/ImageJ (NIH). Signal processing was applied equally across the entire image.

### Adherence and invasion inhibition assay in enteroid monolayers

*Shigella* adherence and invasion inhibition assays were performed using a human colonoid infection assay^46–48^. Colonoid fragments seeded on human collagen IV-coated Transwell inserts (3.0-μm pore-size) were grown as monolayer until confluency confirmed by transepithelial electrical resistance with an epithelial voltohmmeter (EVOM;^2^ World Precision Instruments, Saratosa, FL). Monolayers were differentiated in differentiation medium (DFM)^46–48^ without antibiotics for 5 days. Differentiated colonoid monolayers were inverted and placed in an empty 12-well tissue culture plate. S*. flexneri* 2a or *S. sonnei* were grown in TSB to an OD_600_ of 0.6–0.8 as described above, in the absence or presence of 2.5 mM bile salt deoxycholate (DOC), and resuspended in DFM. An inoculum of 50 µL containing 2.5 × 10^7^ CFU was mixed with 25 µL of heat-inactivated pooled sera from mice immunized with VirGα or AdjuPhos® and added to the basolateral side of colonoid monolayers. In adhesion inhibition assays, the bacteria-immune sera mix was incubated with monolayers for 15 min at 37 °C with 5% CO_2_. In invasion inhibition assays, bacteria grown in the presence of DOC and mixed with mouse immune sera were incubated with monolayers for 1.5 h, and then added 50 µg/mL gentamicin for 30 min to kill extracellular bacteria. Following incubation (in both assays), monolayers were washed three times with PBS, lysed with 0.1% Triton X-100 for 20 min. Recovered bacteria were serially diluted and plated onto TSA plates for CFU enumeration.

### Statistical analysis

All statistical analyses were performed using GraphPad Prism 9.0 (GraphPad Software, La Jolla, CA). Normality tests were performed on continuous data using D’Agostino and Pearson test. Continuous normally distributed data were compared by either unpaired *t* test or one-way ANOVA with Tukey’s multiple comparisons test. Mann–Whitney test and Kruskal–Wallis with Dunn’s multiple comparisons test was used for non-parametric data. Survival curves were compared using a log-rank (Mantel–Cox) test. VE was calculated as [(% death in PBS control animals − % death in vaccinated animals)/% death in PBS control animals] × 100. ROC-AUC analysis was used to identify an antibody threshold level that would predict vaccine protective efficacy. Two-side *P* values were determined, and *P* < 0.05 was considered statistically significant. VirGα amino acid sequence alignments and percent (%) homology were determined using NCBI multiple sequence alignment viewer 1.24.0.

### Reporting summary

Further information on research design is available in the Nature Research Reporting Summary linked to this article.

### Supplementary information

Supplementary Information

REPORTING SUMMARY

## Data Availability

The datasets generated during the current study are available from the corresponding author upon reasonable request.
