# Supplementary material for: Shigella virulence protein VirG is a broadly protective antigen and vaccine candidate
Source: NPJ Vaccines. 2024 Jan 2;9:2. doi: 10.1038/s41541-023-00797-6 (PMC10761965; doi:10.1038/s41541-023-00797-6)
Supplement: Supplementary file 1 — Supplementary Information [file 41541_2023_797_MOESM1_ESM.pdf]

Supplementary Material

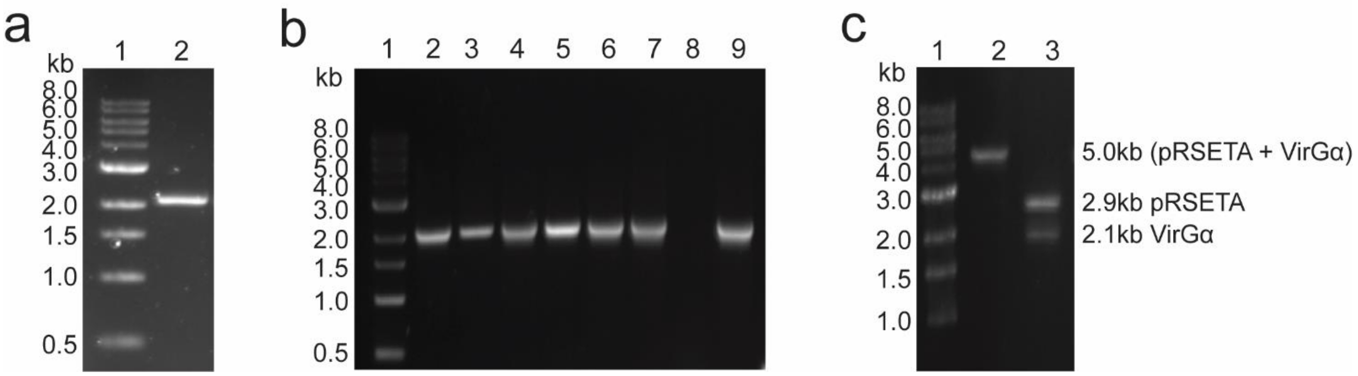

Supplementary Figure 1. Cloning of VirGα. a VirGα gene (2.1 kb) was amplified by polymerase chain reaction (PCR) and run in agarose gel: 2.1 kb VirGα gene (lane 2) and 1 kb DNA ladder (lane 1). **b** agarose gel showing the plasmids bearing the 2.1 kb VirGα gene (lanes 2-6 and 9) and colony without VirGα products (lane 8); 1 kb DNA ladder (lane 1). **c** Plasmids carrying VirGα digested with BamHI featuring 5.0 kb band: pRSETA (2.9 kb) containing the 2.1 kb VirGα gene (lane 2) or digested with BamHI and EcoRI featuring two bands: 2.9 kb pRSETA and 2.1 kb VirGα (lane 3). 1 kb DNA ladder (lane 1).

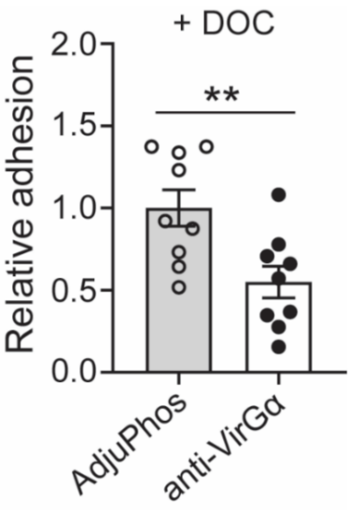

Supplementary Figure 2. VirGα antibodies reduced adherence of *S. sonnei* to human colonoids. *S. sonnei* grown in the presence of DOC and pre-incubated with pooled VirGα mouse antisera were added to the basolateral side of human colonoid monolayers as described in Methods. Relative adherence (or adherence rate) of *S. sonnei* in the presence of anti-VirGα sera was determined compared to that of the negative control (sera from mice that received AdjuPhos® normalized to 1). Assays were performed in at least quadruplicate wells, and two independent experiments were conducted. Data represents mean from replicate wells ± SEM; ** $p < 0.01$, compared to the AdjuPhos® control by *t*-test.

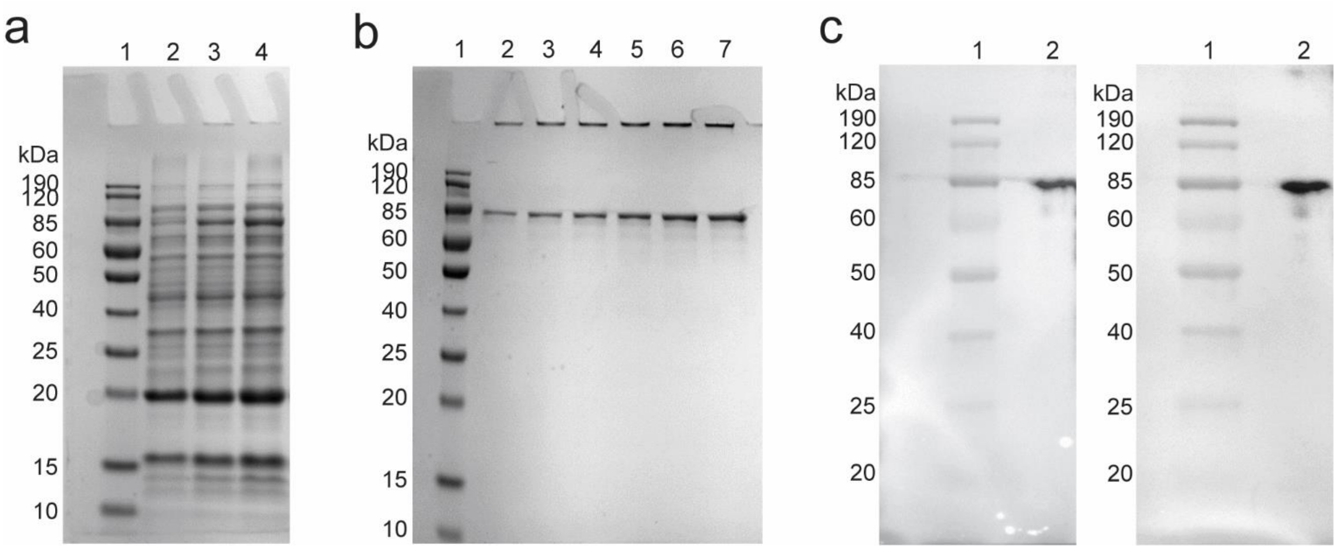

Supplementary Figure 3. Images used to generate Fig 1b-d, showing SDS-PAGE gels and Western blot. Uncropped and unprocessed images corresponding to Fig 1b **(a)**, Fig 1c **(b)** and Fig 1d **(c)**.
