# Supplementary material for: Shigella virulence protein VirG is a broadly protective antigen and vaccine candidate
Source: NPJ Vaccines. 2024 Jan 2;9:2. doi: 10.1038/s41541-023-00797-6 (PMC10761965; doi:10.1038/s41541-023-00797-6)
Supplement: Supplementary file 2 — REPORTING SUMMARY [file 41541_2023_797_MOESM2_ESM.pdf]

nature portfolio

Corresponding author(s): Marcela F Pasetti

Last updated by author(s): Nov 14, 2023

Reporting Summary

Nature Portfolio wishes to improve the reproducibility of the work that we publish. This form provides structure for consistency and transparency in reporting. For further information on Nature Portfolio policies, see our Editorial Policies and the Editorial Policy Checklist.

Statistics

For all statistical analyses, confirm that the following items are present in the figure legend, table legend, main text, or Methods section.

n/a	Confirmed	
☐	☒	The exact sample size (n) for each experimental group/condition, given as a discrete number and unit of measurement
☐	☒	A statement on whether measurements were taken from distinct samples or whether the same sample was measured repeatedly
☐	☒	The statistical test(s) used AND whether they are one- or two-sided<br>*Only common tests should be described solely by name; describe more complex techniques in the Methods section.*
☒	☐	A description of all covariates tested
☐	☒	A description of any assumptions or corrections, such as tests of normality and adjustment for multiple comparisons
☐	☒	A full description of the statistical parameters including central tendency (e.g. means) or other basic estimates (e.g. regression coefficient) AND variation (e.g. standard deviation) or associated estimates of uncertainty (e.g. confidence intervals)
☐	☒	For null hypothesis testing, the test statistic (e.g. F, t, r) with confidence intervals, effect sizes, degrees of freedom and P value noted<br>*Give P values as exact values whenever suitable.*
☒	☐	For Bayesian analysis, information on the choice of priors and Markov chain Monte Carlo settings
☒	☐	For hierarchical and complex designs, identification of the appropriate level for tests and full reporting of outcomes
☐	☒	Estimates of effect sizes (e.g. Cohen's d, Pearson's r), indicating how they were calculated

Our web collection on statistics for biologists contains articles on many of the points above.

Software and code

Policy information about availability of computer code

Data collection	- Confocal imaging was conducted using the Leica MICA microscope and Images were collated using FIJI/ImageJ software (NIH).<br>- Multiskan FC ELISA reader with SkanIt software was used to read microtiter plate and analyze ELISA data.<br>- ELISPOT plates were scanned and spots were counted using a CTL ImmunoSpot® Analyzers along with ImmunoSpot® Software (ImmunoSpot®, CTL).<br>- Colony counts were enumerated using NIST Integrated Colony Enumerator (NICE) software, and an Excel-based software program, Opsotiter, was used to determine SBA titers.
Data analysis	GraphPad Prism 9.0 (GraphPad Software, La Jolla, CA) was used in the data analysis.

For manuscripts utilizing custom algorithms or software that are central to the research but not yet described in published literature, software must be made available to editors and reviewers. We strongly encourage code deposition in a community repository (e.g. GitHub). See the Nature Portfolio guidelines for submitting code & software for further information.

Data

Policy information about availability of data

All manuscripts must include a data availability statement. This statement should provide the following information, where applicable:

- Accession codes, unique identifiers, or web links for publicly available datasets
- A description of any restrictions on data availability
- For clinical datasets or third party data, please ensure that the statement adheres to our policy

The datasets generated during the current study are available from the corresponding author upon reasonable request.

Research involving human participants, their data, or biological material

Policy information about studies with human participants or human data. See also policy information about sex, gender (identity/presentation), and sexual orientation and race, ethnicity and racism.

Reporting on sex and gender	Humans are not included in the study.
Reporting on race, ethnicity, or other socially relevant groupings	Humans are not included in the study.
Population characteristics	Humans are not included in the study.
Recruitment	Humans are not included in the study.
Ethics oversight	Humans are not included in the study.

Note that full information on the approval of the study protocol must also be provided in the manuscript.

Field-specific reporting

Please select the one below that is the best fit for your research. If you are not sure, read the appropriate sections before making your selection.

☒ Life sciences ☐ Behavioural & social sciences ☐ Ecological, evolutionary & environmental sciences

For a reference copy of the document with all sections, see nature.com/documents/nr-reporting-summary-flat.pdf

Life sciences study design

All studies must disclose on these points even when the disclosure is negative.

Sample size	Adult BALB/c mice (10-20 per group) were immunized with VirGα in order to achieve statistically significance. The group sizes were determined based on our past experience of similar experiments and the minimal number needed to establish statistically significant protection data when comparing among vaccinated groups and control groups, with a statistical power of > 80%. Sample size was also determined based on the number typically used in literature to determine immune responses and protective efficacy post-infection.
Data exclusions	No data were excluded from analysis.
Replication	Data were generated from at least two independent experiments.
Randomization	Animals were randomly allocated to different groups in this study.
Blinding	The laboratory personnel who did the immunization, challenge and follow-up monitoring was not blinded. Serology and functional analyses were performed by blinded operators.

Reporting for specific materials, systems and methods

We require information from authors about some types of materials, experimental systems and methods used in many studies. Here, indicate whether each material, system or method listed is relevant to your study. If you are not sure if a list item applies to your research, read the appropriate section before selecting a response.

Materials & experimental systems

n/a	Involved in the study	
☐	☒	Antibodies
☒	☐	Eukaryotic cell lines
☒	☐	Palaeontology and archaeology
☐	☒	Animals and other organisms
☒	☐	Clinical data
☒	☐	Dual use research of concern
☒	☐	Plants

Methods

n/a	Involved in the study	
☒	☐	ChIP-seq
☒	☐	Flow cytometry
☒	☐	MRI-based neuroimaging

Antibodies

Antibodies used	- Horseradish peroxidase (HRP)-labeled goat anti-mouse IgG (Catalogue # 5220-0460, KPL SeraCare, Gaithersburg, MD).<br>- Mouse IgA/IgG double-color ELISpot assay kit [Catalogue # mIgGIgA-DCE-1M/2, ImmunoSpot® ELISpot Kit, Cellular Technology Limited (CTL)]; anti-mouse IgA/IgG (included in the ImmunoSpot® ELISpot Kit, C.T.L).<br>- Goat anti-mouse IgG AF555-labeled conjugate secondary antibody (Catalogue # A32727, Thermo Fisher Scientific)
Validation	We used commercially available secondary antibodies; quality control and validations for use was conducted by vendor and can be found from each vendor's website using the specific catalog number.

Animals and other research organisms

Policy information about studies involving animals; ARRIVE guidelines recommended for reporting animal research, and Sex and Gender in Research

Laboratory animals	Adult female BALB/c mice, 6-8 weeks-old, purchased from Charles River Laboratories (Wilmington, MA) were used for in vivo immunization experiments.
Wild animals	No wild animals were used in this study.
Reporting on sex	We have traditionally evaluated Shigella vaccines in female mice; this has also been standard in the literature. For consistency, female mice were used in these initial experiments to select the best vaccine dosage, conditions of immunization and challenge. Further protection testing will include adult female and male mice.
Field-collected samples	The study did not involve samples collected from the field.
Ethics oversight	All animal studies and procedures were approved by the University of Maryland School of Medicine Institutional Animal Care and Use Committee (IACUC) and conducted in accordance with guidelines from the "Guide for the Care and Use of Laboratory Animals" of the National Institutes of Health (NIH). Every effort possible was made to minimize pain and distress of the animals.

Note that full information on the approval of the study protocol must also be provided in the manuscript.

Plants

Seed stocks	Plants are not used in this study
Novel plant genotypes	Plants are not used in this study
Authentication	Plants are not used in this study
